# A receptor-like kinase gene (*GbRLK*) from *Gossypium barbadense* enhances salinity and drought-stress tolerance in *Arabidopsis*

**DOI:** 10.1186/1471-2229-13-110

**Published:** 2013-08-06

**Authors:** Jun Zhao, Yulong Gao, Zhiyuan Zhang, Tianzi Chen, Wangzhen Guo, Tianzhen Zhang

**Affiliations:** 1 National Key Laboratory of Crop Genetics & Germplasm Enhancement, MOE Hybrid Cotton R&D Engineering Research Center, Nanjing Agricultural University, Nanjing 210095 Jiangsu Province, China

**Keywords:** *Gossypium barbadense*, Receptor-like protein kinase, Abscisic acid, *Arabidopsis thaliana*, Abiotic stress tolerance, Transgene

## Abstract

**Background:**

Cotton (*Gossypium* spp.) is widely cultivated due to the important economic value of its fiber. However, extreme environmental degradation impedes cotton growth and production. Receptor-like kinase (RLK) proteins play important roles in signal transduction and participate in a diverse range of processes in response to plant hormones and environmental cues. Here, we introduced an RLK gene (*GbRLK*) from cotton into *Arabidopsis* and investigated its role in imparting abiotic stress tolerance.

**Results:**

*GbRLK* transcription was induced by exogenously supplied abscisic acid (ABA), salicylic acid, methyl jasmonate, mock drought conditions and high salinity. We cloned the promoter sequence of this gene *via* self-formed adaptor PCR. Sequence analysis revealed that the promoter region contains many *cis*-acting stress-responsive elements such as ABRE, W-Box, MYB-core, W-Box core, TCA-element and others. We constructed a vector containing a 1,890-bp sequence in the 5′ region upstream of the initiation codon of this promoter and transformed it into *Arabidopsis thaliana*. GUS histochemical staining analysis showed that *GbRLK* was expressed mainly in leaf veins, petioles and roots of transgenic *Arabidopsis*, but not in the cotyledons or root hairs. *GbRLK* promoter activity was induced by ABA, PEG, NaCl and *Verticillium dahliae.* Transgenic *Arabidopsis* with constitutive overexpression of *GbRLK* exhibited a reduced rate of water loss in leaves *in vitro*, along with improved salinity and drought tolerance and increased sensitivity to ABA compared with non-transgenic Col-0 *Arabidopsis*. Expression analysis of stress-responsive genes in *GbRLK Arabidopsis* revealed that there was increased expression of genes involved in the ABA-dependent signaling pathway (*AtRD20, AtRD22* and *AtRD26*) and antioxidant genes (*AtCAT1, AtCCS, AtCSD2* and *AtCSD1*) but not ion transporter genes (*AtNHX1, AtSOS1*).

**Conclusions:**

*GbRLK* is involved in the drought and high salinity stresses pathway by activating or participating in the ABA signaling pathway. Overexpression of *GbRLK* may improve stress tolerance by regulating stress-responsive genes to reduce water loss. *GbRLK* may be employed in the genetic engineering of novel cotton cultivars in the future. Further studying of *GbRLK* will help elucidate abiotic stress signaling pathways.

## Background

Cotton is an economically important crop; cotton fiber serves as the primary raw material for the textile industry. However, as environmental degradation continues, cotton is becoming increasingly subjected to extreme drought, high salinity and temperature extremes, each of which can impede plant growth and production [1]. Plant molecular responses to abiotic stresses involve interactions and crosstalk between many molecular pathways. Hormones are important regulators of plant responses to abiotic stress. One of the most important stress-responsive plant hormones is abscisic acid (ABA). ABA is one of the most important phytohormones and is a key regulator of plant responses to various environmental stresses [1]. ABA plays an important role in the tolerance of plants to drought and high salinity, and the mechanisms underlying ABA activity have been studied extensively for several decades [2-4]. The main functions of ABA include regulating plant water balance and increasing osmotic stress tolerance by inducing seed dormancy, delaying seed germination and promoting stomatal closure [1]. To date, many stress-responsive genes have been identified; many of these genes are regulated by ABA. Recently, several ABA-deficient mutants have been reported in *Arabidopsis*, tobacco, tomato and maize [5]. Without stress treatment, the growth of these mutants is comparable to that of wild-type plants. Under drought stress, ABA-deficient mutants exhibit poor growth or even death [6,7]. Studying these mutants has revealed the presence of two major stress-related pathways, i.e., ABA-dependent and ABA-independent gene pathways [2]. For cotton, high throughput screening techniques such as transcriptome have been used to study the adaptability in drought. Payton et al. [8] found that the majority of stress-responsive transcripts had tissue-specific expression patterns using root and leaf under the water-deficit stress. Genome-wide transcriptomic analysis of cotton under drought stress revealed significant down- regulation of genes and pathways involved in fiber elongation and up-regulation of defense responsive genes under drought stress [9]. Using two accessions *G. herbaceum* with different tolerant to osmotic stress, Ranjan et al. [10] found that drought tolerance for cotton was not because of a single molecular reason but was rather due to several unique mechanisms.

Plants can receive and recognize signals and stimuli from the environment through various classes of receptors, including receptor-like kinase (RLK) proteins, which play important roles in signal transduction pathways [11]. RLK proteins kinases are encoded by a multigene family, which represents one of the largest gene families in the *Arabidopsis* genome, comprising at least 610 members [12], with approximately 1,131 members in rice [13]. The size variations of the RLK families have been affected by many factors such as natural and artificial selection, living environments, genome size variation, polyploidization [14]. RLKs are characterized by the presence of a signal sequence, an amino-terminal domain with a transmembrane region and a carboxyl-terminal kinase domain [15,16]. Since the discovery of the first *RLK* many years ago, substantial efforts have been devoted to examining a few receptor-kinase genes. *RLK*s participate in a diverse range of processes in response to plant hormones and environmental cues and are involved in the regulation of many physiological changes in plants such as self-incompatibility, endosperm and pollen development, brassinosteroid sensing, anthoptosis and stress and disease resistance [11,12]. Like *Arabidopsis* brassinosteroid insensitive 1 (*BRI1*) [17,18], *CLAVATA1*[19] and *HAESA*[20] control plant growth and development under normal growth conditions. Tomato *Pto*[21], rice *Xa21*[22] and *Arabidopsis FLS2*[23] take part in the plant defense response and in plant-microbe interactions. Many researches suggested that RLKs can play an important role in optimizing plant responses to drought stress [24-26]. *Arabidopsis ARCK1*[27], *GHR1*[28] and *RPK1*[29] have been implicated in controlling plant abiotic stress tolerance. SnRK2s (SNF1-related protein kinase) is a family of ABA-activated protein kinases [3,6,30]. In *Arabidopsis*, three members of this family, SRK2D/SnRK2.2, SRK2E/OST1/SnRK2.6 and SRK2I/SnRK2.3, regulate ABA signaling both positively and globally [6]. Rice receptor-like kinase *OsSIK1* was also proved to improve drought and salt stress tolerance [31].

Cotton genes encoding protein kinases have been reported, but these genes function mainly in the regulation of cotton fiber development. To our knowledge, RLK genes associated with cotton abiotic stress have not previously been documented. The RLK gene (*GbRLK*) identified in this study was previously identified among a group of expressed resistance gene analogs that are induced by *Verticillium dahliae* (VD) in the disease-resistant cotton *Gossypium barbasense* cv. Hai7124 (L447 published in Gao *et al*., 2006) [32]. Transgenic cotton and *Arabidopsis* plants that overexpress *GbRLK* show increased resistance to VD. In the current study, we cloned the promoter region of *GbRLK* and analyzed the expression of *GbRLK* in response to abiotic stress. The results of this study suggest that *GbRLK* can be induced by ABA, drought stress and salinity. The overexpression of *GbRLK* in *Arabidopsis* increased plant tolerance to drought and high salinity and increased sensitivity to ABA. The analysis of this kinase gene will help elucidate the molecular basis of RLK protein function in abiotic stress tolerance in cotton. This study also provides a candidate gene for further research on ABA signaling pathways in cotton and other plants subjected to abiotic stress.

## Results

### Phylogenetic relationships between GbRLK and other kinase proteins

**Figure 1:**
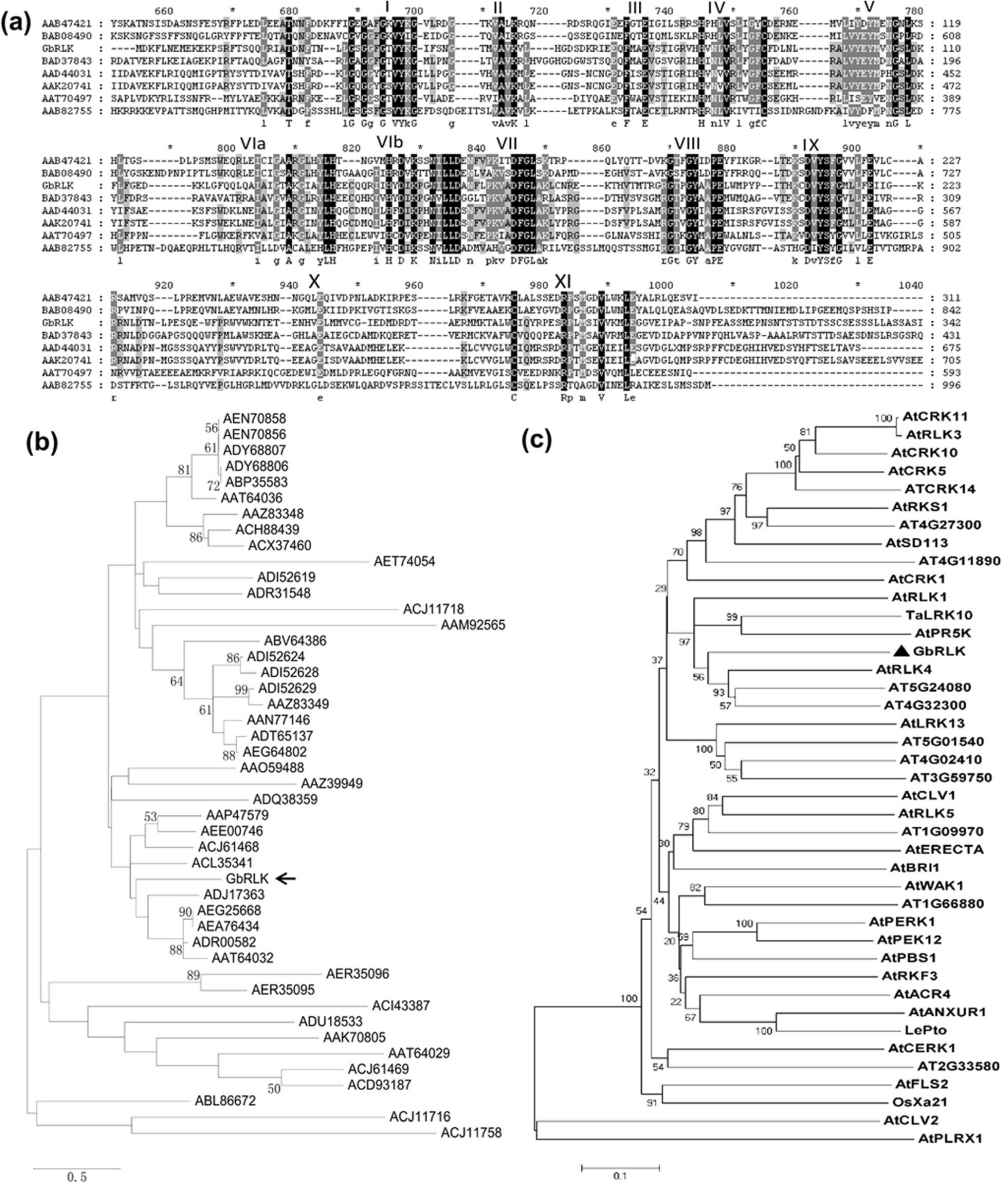
**Phylogenetic analysis of *GbRLK*. (a)** Alignment of deduced amino acid sequences of *GbRLK* and other kinases. (AAB47421, *Pto* from tomato; BAB08490, protein kinase from *Arabidopsis*; AAT70497, protein kinase from peach; BAD37843, protein kinase from rice; AAD44031, *PK3* from barley; AAK20741, *TAK33* from barley; AAB82755, *Xa21* from rice). **(b)** Phylogenetic relationships between GbRLK and other kinase proteins from *Gossypium hirsutum* and *Gossypium barbadense*. **(c)** Phylogenetic relationships between GbRLK and other reported receptor-like kinase proteins from the eudicot *Arabidopsis thaliana* (AtANXUR1, AEE74120; AtPBS1, AED91858; AtCERK1, AEE76532; AtPR5K, AED94289; AtACR4, AEE79920; AtWAK1, AEE30079; AtRKF3, AEC10923; AtBRI1, AEE87069; AtERECTA, AEC07825; AtCLV1, AEE35762; AtRLK5, AEE85494; AtPERK1, AEE76920; AtPEK12, AEE30401; AtCRK1, AEE29801; AtSD113, AEE28721; AtRKS1, AEE28720; AtCRK5, EFH43988; AtCRK10, AEE84719; AtRLK3, CAA09731; AtFLS2, AED95370; AtLRK13, AEE78027; AtRLK1, AED97394; AtRLK4, BAC42412; AT4G27300; AT4G11890; AT5G24080; AT4G32300; AT5G01540; AT4GO2410; AT3G59750; AT1G09970; AT1G66880; AT2G33580) and some representative members of *Oryza sativa* (Xa21, AAB82755), *Lycopersicon esculentum* (LePTO, BAB08490), and *Triticum aestivum* (TaLRK10, AAC49629). The phylogenetic tree was constructed using the maximum likelihood method in MEGA 5.0. Numbers on internal nodes are the percentage bootstrap support values (1000 re-sampling); only values exceeding 50% are shown.

A functional *GbRLK* gene [NCBI accession number KC422776], which was induced by *Verticillium dahliae,* was previously identified among a group of expressed resistance gene analogs from the disease-resistant cotton *Gossypium barbasense* cv. Hai7124 using RACE [32]. The cDNA was 1536-bp in length and the predicted ORF starts at nucleotide 330 and ends at nucleotide 1409, encoding a predicted protein of 359 amino acids. BLASTx searches revealed that the predicted product shared 68% identity and 75% similarity with the soybean G-type lectin S-receptor-like serine/threonine -protein kinase (E value: 0), 56% identity and 72% similarity with the rice *PK*3 (E value: 1e^-98^), 48% and 65% (E value: 4e^-73^) with the wheat *TAK*33, and 48% and 64% (E value: 2e^-71^) with the barley receptor-like protein kinase, respectively. The protein possessed intact signature features of protein tyrosine kinases, and contained conserved motifs characteristic of I-XI of receptor-like protein kinases, which are possessed in the majority of protein kinases. Multiple sequence alignments using BLASTx revealed that the protein shared homology in other regions with protein kinases from various other crop species (Figure 1a). We therefore conclude that this protein may belong to the receptor-like protein kinase family and be a member of the *RLK* gene family. This gene was thereafter designated as *G. barbadense* receptor-like protein kinase (*GbRLK*). *GbRLK* contains an ATP binding site (residues 32 to 40), a D activation site (at residue 150), four N-glycosylation sites, four N-myristoylation sites, three C terminal phosphorylation sites, one amidation site, four tyrosine kinase II phosphorylation sites, and a membrane-spanning region (residues 28–46).

Currently the NCBI databases contain 46 cotton sequences predicted to encode protein kinases, and among them, three (AAP47579, AA059488, AAN77146) were known to be involved in fiber development. The functions of the other sequences were unknown. The phylogenetic relationship between the GbRLK protein and the other known cotton protein kinases via the construction of phylogenetic tree revealed that GbRLK protein was most closely related evolutionally to sequences ADJ17363, AEG25668, AEA76434, ADR00582 and AAT64032 that were receptor kinase, somatic embryogenesis receptor-like kinase 1, 2, 3 protein and putative leucine-rich repeat transmembrane protein (Figure 1b).

Phylogenetic analysis was used to investigate the relationship among the GbRLK and other reported receptor-like kinase proteins from eudicot *Arabidopsis thaliana* and *Lycopersicon esculentum* monocot *Oryza sativa*, and *Triticum aestivum*. GbRLK protein was found to cluster together with other reported receptor-like kinase proteins (Figure 1c).

### Temporal and spatial expression analysis of *GbRLK* under abiotic stress

**Figure 2:**
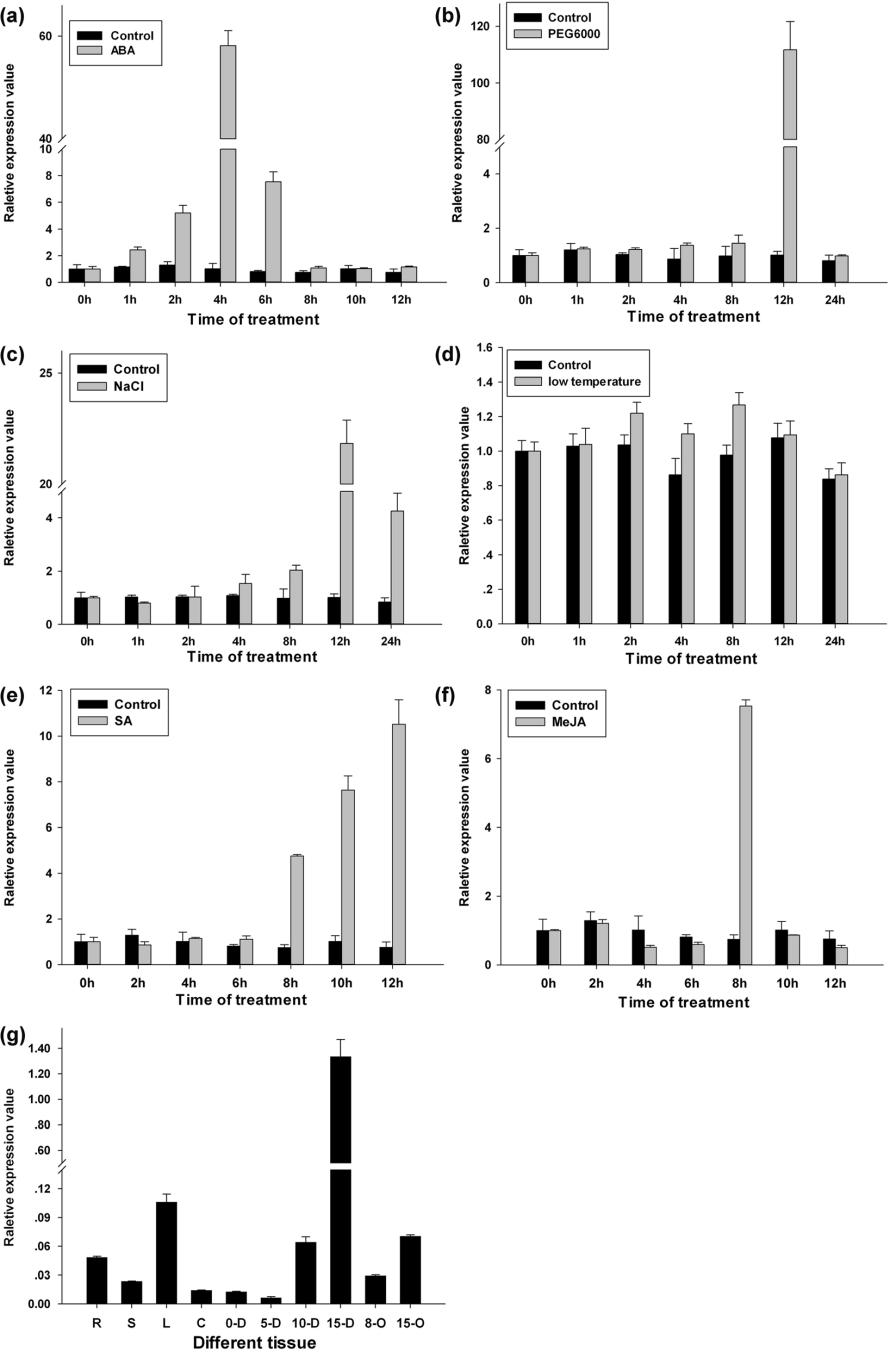
**Analysis of *GbRLK *expression profiles in cotton seedlings in response to different stress treatments.** Expression patterns of *GbRLK* at different time intervals in leaves under ABA (100 μM) treatment **(a)** for 0, 1, 2, 4, 6, 8, 10 and 12 h; drought (20% w/v PEG6000) **(b)**, salinity stress (200 mM NaCl ) **(c)** and cold stress (4°C) **(d)** for 0, 1, 2, 4, 8, 12 and 24 h. The treatment was carried out by spraying the plants with 2 mM SA **(e)** and 100 μM MeJA **(f)** solution at 0, 2, 4, 6, 8, 10 and 12 h. Relative gene expression levels were determined using the 2^-ΔΔCT method. The CT (cycle threshold) values for both the target and internal control genes were means of three technical replicates. ΔCt = Ct target gene-Ct EF1α. ΔΔCT = (CTtarget -CTEF1α) xh - (CTtarget - CTEF1α) 0 h. **(g)** analysis of *GbRLK* expression profiles in different developmental stages and tissue. R: root, S: stem, L: leaf, C: cotyledons, 5-D, 10-D, 15-D represented the 5, 10, 15 days post anthesis of fiber, respectively; 0-D, 8-O, 15-O represented 0, 10, 15 days ovule, respectively. *EF1a* (At5g60390; EF-F/R; Additional file 1: Table S1) from cotton was used as an internal control for normalization of different cDNA samples. Error bars represent standard error of means based on three independent reactions.

Quantitative real-time (qRT-PCR) was used to analyze the expression patterns of *GbRLK* in H7124 leaves exposed to ABA, drought, salinity, SA, MeJA and low temperature. As shown in Figure 2, *GbRLK* was induced in leaves but was differentially expressed in response to SA, MeJA and abiotic stresses. *GbRLK* expression was gradually up-regulated in leaves exposed to 1 to 4 h of ABA treatment; the transcription level of *GbRLK* increased and peaked at 4 h, after which the level of mRNA accumulation gradually declined after 6 to 12 h of ABA treatment (Figure 2a). When the seedlings were treated with PEG6000 (Figure 2b) or NaCl (Figure 2c), the transcription level of *GbRLK* increased rapidly and reached the highest level after 12 h of treatment, followed by a rapid decline, reaching pretreatment levels after 24 h of treatment. The expression of *GbRLK* was not significantly altered under low temperature treatment, suggesting that *GbRLK* expression is not affected by cold stress (Figure 2d). For the SA, the expression of *GbRLK* was induced and up-regulated at 8 h and peaked at 12 h (Figure 2e). When the seedlings were treated with MeJA, the transcription level of *GbRLK* peaked rapidly at 8 h and declined rapidly and reached pretreatment levels at 10 h (Figure 2f).

To further understand the temporal-spatial distribution of *GbRLK* gene, we analyze the expression patterns of *GbRLK* in H7124 different tissue. The results indicated that the *GbRLK* transcripts were difference significant in different tissue. The maximal transcripts of *GbRLK* were detected at 15 DPA (Figure 2g). This implied that the *GbRLK* gene may play a multi-functional role in different development stages.

### Cloning and analysis of the *GbRLK* promoter

To further study the characteristics of *GbRLK* expression, we cloned the promoter sequence of *GbRLK*. The Sefa-PCR generated a 3,227-bp fragment, including a 347-bp region containing previously identified sequences (Additional file 1: Table S1, Additional file 2: Figure S1a). The transcriptional start site and several regulatory elements were predicted using tools including Neural Network Promoter Prediction [33], PlantCARE [34] and PLACE [35]. The 3,227-bp fragment contained many *cis*-acting stress-responsive elements, such as ABRE, W-Box, MYB-core, W-Box core, TCA-element and others (Additional file 3: Table S2).

**Figure 3:**
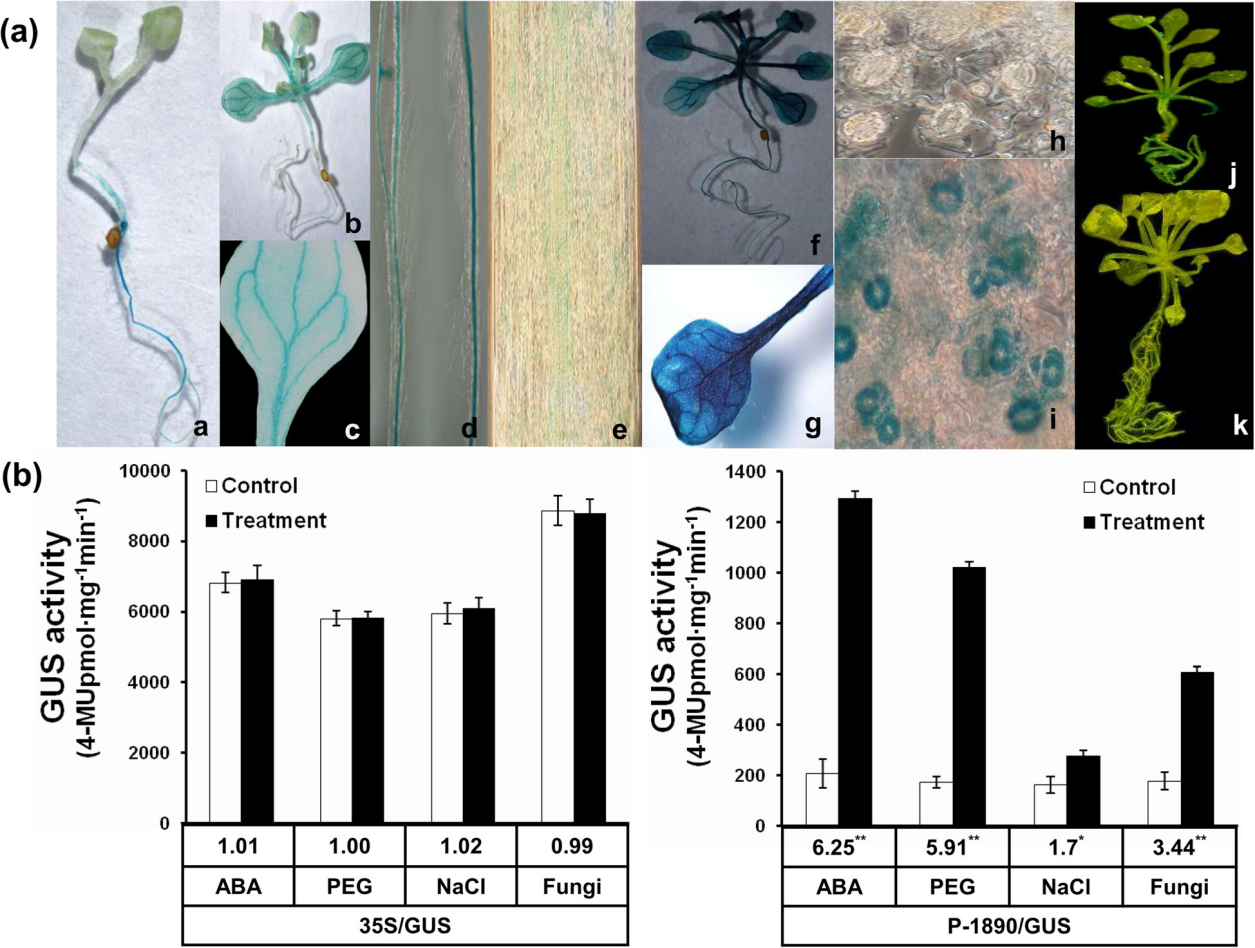
**Histochemical straining and measurement of GUS activity in transgenic *Arabidopsis thaliana*. (a)** a: 3-day-old seedling; b: 7-day-old seedling; c: mature leaf; d: root; e: microscopically observed root; f, g, i: transgenic plants treated with 100 μM ABA for 4 h; h: stomata before treatment with ABA; j: the CaMV 35S (pCAMBIA1301 vector) transformants as the positive control; k: non-transgenic Col-0 as the negative control. **(b)** GUS activity driven by the *GbRLK* promoter in above-ground tissues of transgenic *Arabidopsis* in response to ABA, PEG, NaCl and *Verticillium dahliae*. GUS activity from the CaMV 35S (pCAMBIA1301 vector) transformants served as the control. Data are mean and standard deviations of three replicates. The numbers below the bars indicate the -fold changes in GUS activity. Standard deviations (bars) are indicated. Significance of the changes produced after each treatment was assessed using Student’s t-tests (*P < 0.05, **P < 0.01).

The 1,890-bp fragment (Additional file 2: Figure S1b) on the 5′ region upstream of the initiation codon of the *GbRLK* promoter transferred into *Arabidopsis* to address the regulatory mechanisms employed by the *GbRLK* promoter. The resulting transgenic *Arabidopsis* lines were designated P-1890/GUS. Histochemical staining revealed that *GUS* expression occurred mainly in the vascular bundles (Figure 3a-e) and was strictly confined to the leaf veins (Figure 3a-b), petioles (Figure 3a-c) and roots (Figure 3a-d,e), but not or very low in the cotyledons (Figure 3a-a) or root hairs (Figure 3a-d) of transgenic *Arabidopsis* (Figure 3a). Therefore, the *GbRLK* promoter displayed a tissue-specific expression pattern. This differed from the expression pattern in plants transformed with the cauliflower mosaic virus 35S (CaMV 35S) promoter controlling *GUS,* which served as a positive control. These plants exhibited constitutive *GUS* expression (Figure 3a-j,k).

The expression of *GbRLK* has been shown to respond to stress stimuli; therefore, our next step was to test whether the application of stress stimuli could trigger the expression of the *GbRLK* promoter. The induction of GUS activity was observed in ground tissues of P-1890/GUS transgenic plants upon treatment with ABA, PEG, NaCl or *V. dahlia*. As shown in Figure 2b, the application of ABA, PEG, NaCl or *V. dahlia* activated *GUS* expression in the tissues of P-1890/GUS plants, increasing the GUS activity level by 6.25, 5.91, 1.7 and 3.44-fold, respectively, which differed from that of plants containing *GUS* driven by the 35S (CaMV 35S) promoter (Figure 3b). Interestingly, GUS activity was strongly induced by ABA treatment (Figure 3a-f,g,h,j). Additionally, various stresses rapidly induced increasing transcript level of *GUS* gene (Additional file 2: Figure S1c).

### Overexpression of *GbRLK* improved salt and drought tolerance in transgenic *Arabidopsis*

The 35S::*GbRLK* construct was introduced into *Arabidopsis* ecotype Col-0 and four independent fertile primary *Arabidopsis* transformants were regenerated, and positive transgenic plants were confirmed by PCR detection of *NPT*II and 35S-*GbRLK* (Additional file 4: Figure S2a). These T0 transgenic lines were self-pollinated to produce four homozygous lines, which were designated K-2, K-3, K-5 and K-6. Southern blotting analysis demonstrated that these lines arose from independent transformants, and each line carried between one and three copies of the *GbRLK* gene (Additional file 4: Figure S4b). The results of qRT-PCR revealed that *GbRLK* was expressed in all four transgenic *Arabidopsis* lines, with the highest level of expression in the K-2 line (Additional file 4: Figure S2c). The four transgenic lines showed no morphological aberrations through the T5 generation. No consistent difference in fresh weight was observed between the control and transgenic *Arabidopsis* lines (Additional file 4: Figure S2d).

**Figure 4:**
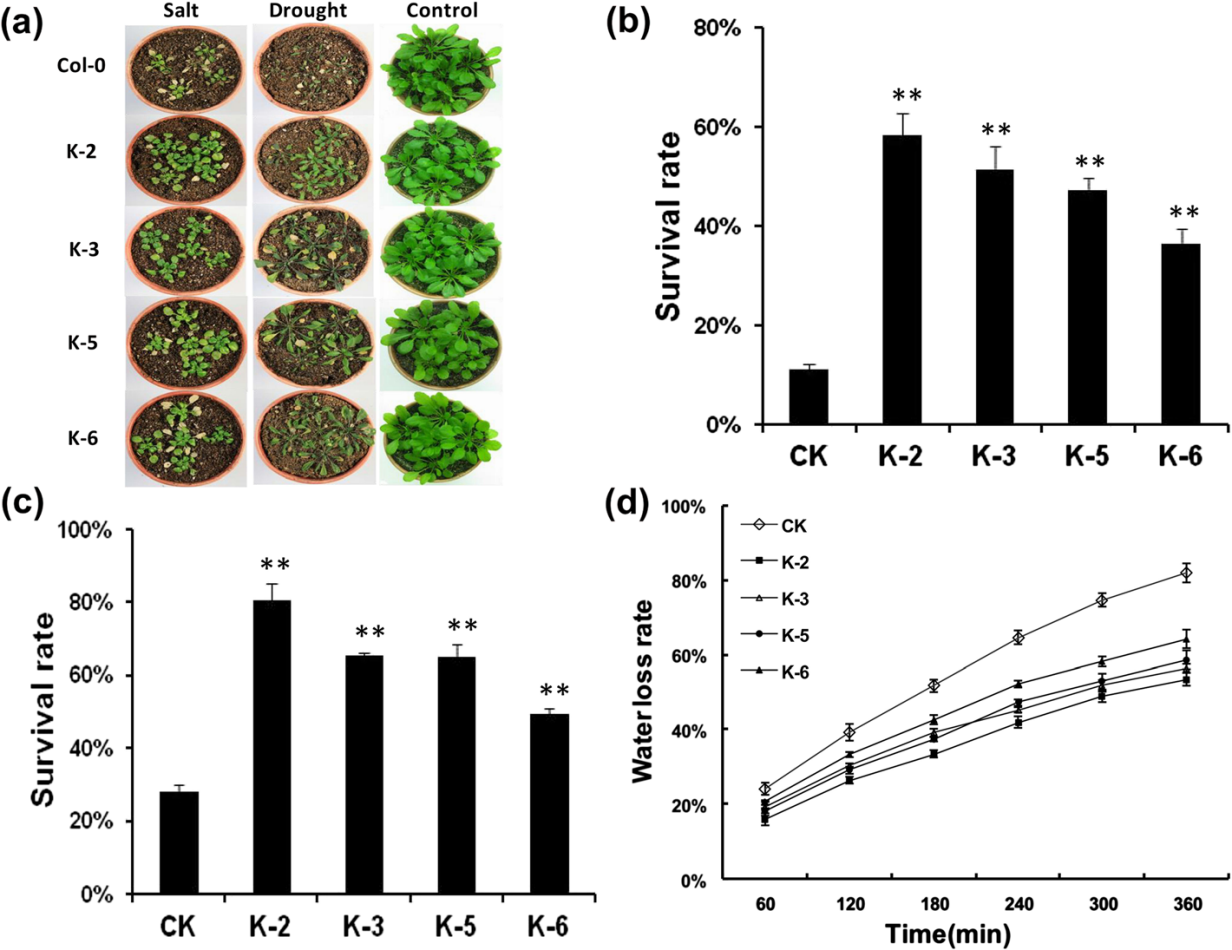
**Analysis of wild-type and 35S::*GbRLK *transgenic *Arabidopsis *lines subjected to salt and drought treatment. (a)** The response of transgenic *Arabidopsis* to salt and drought stress. Wild-type (Col-0) and transgenic *Arabidopsis* were grown in pots containing soil mixture (rich soil: vermiculite = 2:1, v/v) and maintained in a growth chamber at 22°C on a 16-h light/8-hr dark regimen. For drought stress treatment, the photograph was taken after withholding water for 42 days. For salt stress treatment, the photograph was taken after treatment with a 150 mM NaCl solution for 35 days at 22°C. The experiments were repeated three times with similar results. **(b)** and **(c)** represent the survival ratios of transgenic *Arabidopsis* lines and Col-0 (CK) under drought-stress treatment for 42 days and salt-stress treatment for 35 days, respectively. **(d)** Water loss rate in transgenic and non-transgenic plants; standard deviations (bars) are indicated. Comparisons were made between wild-type and individual transgenic lines under control or drought-stress conditions using the paired t-test. ** indicates significant differences compared with the control at P < 0.01.

The analysis of the transgenic plants tolerance to drought revealed that the wild-type plants showed more severe symptoms than the transgenic plants (Figure 4a). Only 10% of the WT plants survived to maturity, whereas 58%, 51%, 47% and 36% of the transgenic plants survived in lines K-2, K-3, K-5 and K-6, respectively (Figure 4b). For analysis of high-salt tolerance, 3-week-old seedlings of transgenic and wild-type *Arabidopsis* grown in pots (with 4–5 leaves per plant ) were irrigated with a solution containing 200 mM NaCl once a week for 5 weeks. As shown in Figure 3a, both the transgenic and wild-type plants showed etiolation and yellowing, but the leaves of the WT plants wilted**,** and some of the WT plants died. The survival rate was approximately 27% in the WT compared to 80%, 65%, 64% and 49%, respectively in the four transgenic lines (Figure 4c). Additionally, there were also significant differences in fresh weight (FW), rosette diameter, anthocyanin accumulation, chlorophyll content and level of leaf chlorosis between the transgenic and the WT plants suffered drought and salt stresses (Tables 1 and 2). It is concluded that the transgenic *Arabidopsis* lines are more tolerant to drought and salt stresses compared to the WT, and among these transgenic lines, K-2 showed the highest tolerance, consistent with the expression level of the *GbRLK*. Overall, the WT plants showed more serious symptoms than the transgenic plants.

**Table 1:** **The symptoms of the transgenic*Arabidopsis*and WT suffered drought stress**

	**FW(g)**	**Rosette diameter(cm)**	**Content of chlorophyll a (nmol/g)**	**Content of chlorophyll b (nmol/g)**	**Content of anthocyanin (nmol/g)**
**N**	0.237 ± 0.07	5.24 ± 0.60	0.157 ± 0.05	0.135 ± 0.03	27.522 ± 3.80
**K- 2**	1.407 ± 0.08^**^	7.72 ± 1.34^**^	0.755 ± 0.09^**^	0.338 ± 0.05^**^	16.006 ± 2.29^**^
**K- 6**	0.664 ± 0.08^**^	6.90 ± 1.10^*^	0.492 ± 0.09^**^	0.249 ± 0.06^*^	18.141 ± 2.89^**^
**K- 3**	0.735 ± 0.12^**^	7.74 ± 0.64^**^	0.654 ± 0.09^**^	0.296 ± 0.02^**^	17.295 ± 1.13^**^
**K- 5**	0.821 ± 0.06^**^	7.85 ± 0.94^**^	0.684 ± 0.11^**^	0.318 ± 0.07^**^	17.326 ± 1.83^**^
**P**	1.886 ± 0.10	9.73 ± 0.58	0.967 ± 0.06	0.406 ± 0.06	9.650 ± 1.84

The data were acquired from the 3 week old plants treated by withholding irrigation for 3 weeks. Data represent the mean ± SE (n ≥ 15); similar results were obtained from three independent experiments. N: wild-type Col-0 suffered drought stress, and P: non-treatment wild-type Col-0. Significance of the phenotypic results was assessed using Student’s t tests (*P < 0.05, **P < 0.01).

**Table 2:** **The symptoms of the transgenic*Arabidopsis*and WT suffered salt stress**

	**FW(g)**	**Rosette diameter(cm)**	**Content of chlorophyll a (nmol/g)**	**Content of chlorophyll b (nmol/g)**	**Content of anthocyanin (nmol/g)**	**Rate of chlorosis (150 mM)**	**Rate of chlorosis (100 mM)**	**Germination rates (300 mM)**
**N**	0.190 ± 0.02	2.45 ± 0.39	0.211 ± 0.05	0.151 ± 0.02	21.220 ± 1.45	90.2% ± 2.9%	59.2% ± 3.2%	3.3% ± 0.9%
**K- 2**	0.588 ± 0.09^**^	5.40 ± 0.65^**^	0.852 ± 0.06^**^	0.365 ± 0.07^**^	15.116 ± 3.14^**^	58.1% ± 1.4%^**^	20.0% ± 4.7%^**^	33.3% ± 1.6%^**^
**K- 6**	0.387 ± 0.03^**^	4.48 ± 0.49^**^	0.557 ± 0.72^**^	0.269 ± 0.05^**^	17.428 ± 2.38^*^	76.3% ± 2.2%^*^	48.3% ± 1.9%^*^	23.3% ± 2.1%^**^
**K- 3**	0.303 ± 0.05^**^	4.29 ± 0.29^**^	0.691 ± 0.05^**^	0.346 ± 0.03^**^	16.356 ± 1.30^*^	63.3% ± 2.6%^**^	32.5% ± 3.2%^**^	30.0% ± 2.5%^**^
**K- 5**	0.397 ± 0.05^**^	4.90 ± 0.48^**^	0.604 ± 0.12^**^	0.325 ± 0.09^**^	16.264 ± 1.02^*^	66.7% ± 7.2%^**^	47.5% ± 5.7%^*^	26.7% ± 1.9%^**^
**P**	1.886 ± 0.10	9.73 ± 0.58	0.967 ± 0.06	0.406 ± 0.06	9.650 ± 1.84	--	--	1

The data of FW, rosette diameter, content of chlorophyll and anthocyanin were acquired from the 3 week old plants treated by submerging the roots of the plants in 200 mM NaCl solution for 3 weeks. The data of rate of chlorosis and germination rates were acquired from plants grown on MS medium containing different concentration NaCl for 2 weeks. Data represent the mean ± SE (n ≥ 15); similar results were obtained from three independent experiments. N: wild-type Col-0 suffered drought stress, and P: non-treatment wild-type Col-0. Significance of the phenotypic results was assessed using Student’s t tests (*P < 0.05, **P < 0.01).

Furthermore, assays of water loss from detached leaves showed that the transgenic plants lost water much more slowly than the WT plants. At 360 min, the water loss rates were 53.5%, 56.4%, 58.6% and 64.2% in the transgenic lines K-2, K-3, K-5 and K-6, respectively, compared to 81.9% in the WT plants (Figure 4d). These results suggest that the overexpression of *GbRLK* reduces water loss and increases drought tolerance in transgenic plants. Therefore, *GbRLK* may function as a positive regulator of plant responses to drought stress.

### Transgenic *Arabidopsis* plants were more sensitive to ABA than WT

**Figure 5:**
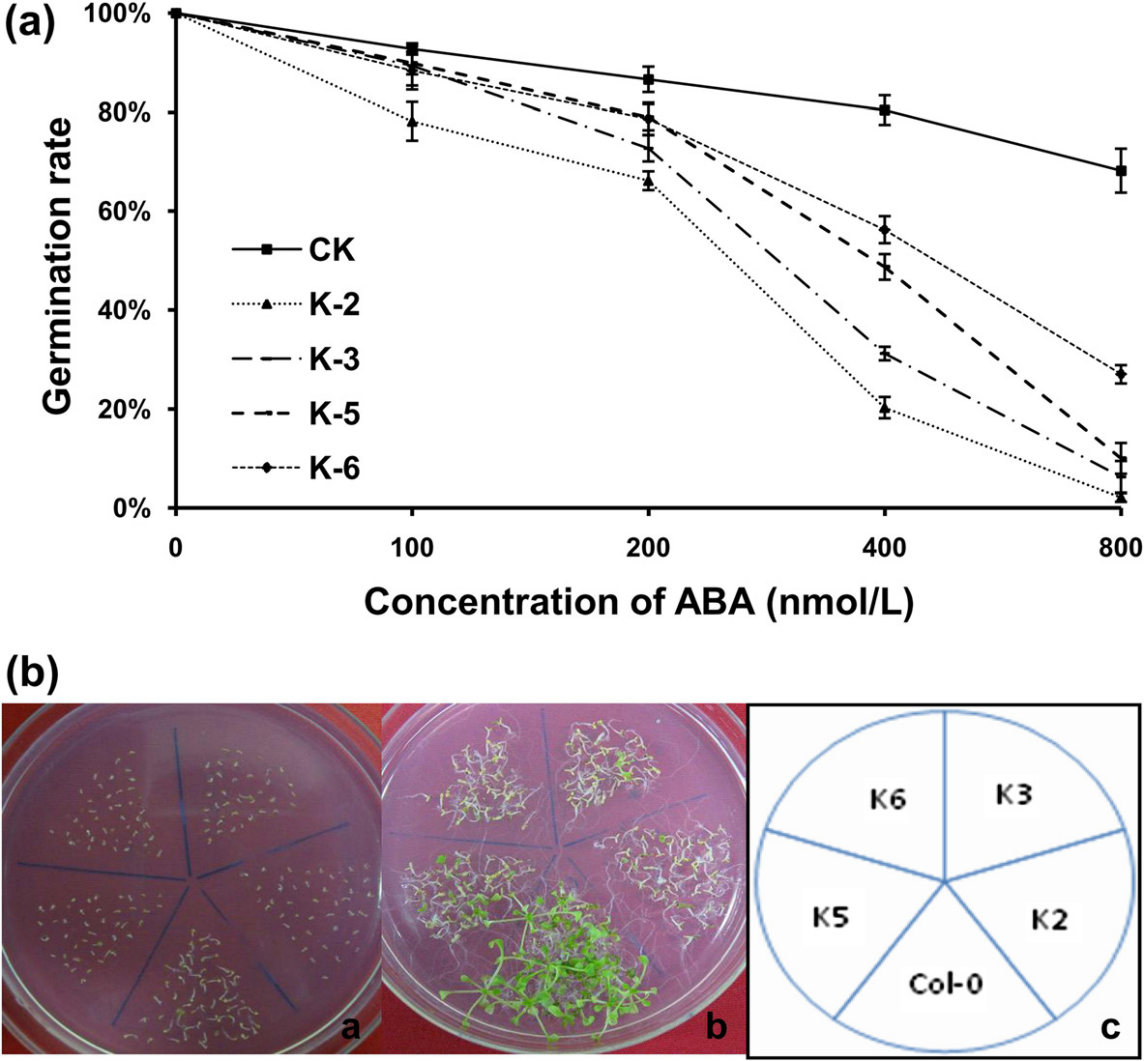
**Analysis of *35S::GbRLK *transgenic lines subjected to ABA treatment. (a)** Germination ratios of transgenic lines and Col-0 on MS medium supplemented with 0, 25, 50, 100, 200, 400 and 800 nmol/L ABA. Standard deviations (bars) are indicated. **(b)** Analysis of the sensitivity of *35S::GbRLK* transgenic lines to MS medium supplemented with 100 nmol/L ABA at different time points. The photographs were taken after 7 and 14 days of treatment in a and b , respectively.

To examine changes in the response of *GbRLK* transgenic plants to ABA, we investigated the ABA sensitivity of the transgenic plant. Under ABA treatment, the germination rates of the transgenic lines were greatly reduced (Figure 5a). When grown on MS medium containing 100 nmol ABA for 1, 2 and 3 weeks, the transgenic plants exhibited many abnormal phenotypes such as dwarfing, etiolation and malformation (Figure 5b). Furthermore, root elongation in the 35S::*GbRLK* transgenic lines was hypersensitive to ABA (data not shown). These results demonstrate that ABA suppresses seed germination, root elongation and plant growth rates more strongly in *GbRLK* transgenic plants than in the WT. This indicates that ABA application activates the GbRLK protein or triggers other responses in transgenic plants. In addition, these results also suggest that the GbRLK protein activates or participates in ABA-dependent signal transduction cascades or plays an important regulatory role in ABA and stress responses.

### *GbRLK* overexpression altered the expression pattern of stress-responsive genes in transgenic *Arabidopsis*

**Figure 6:**
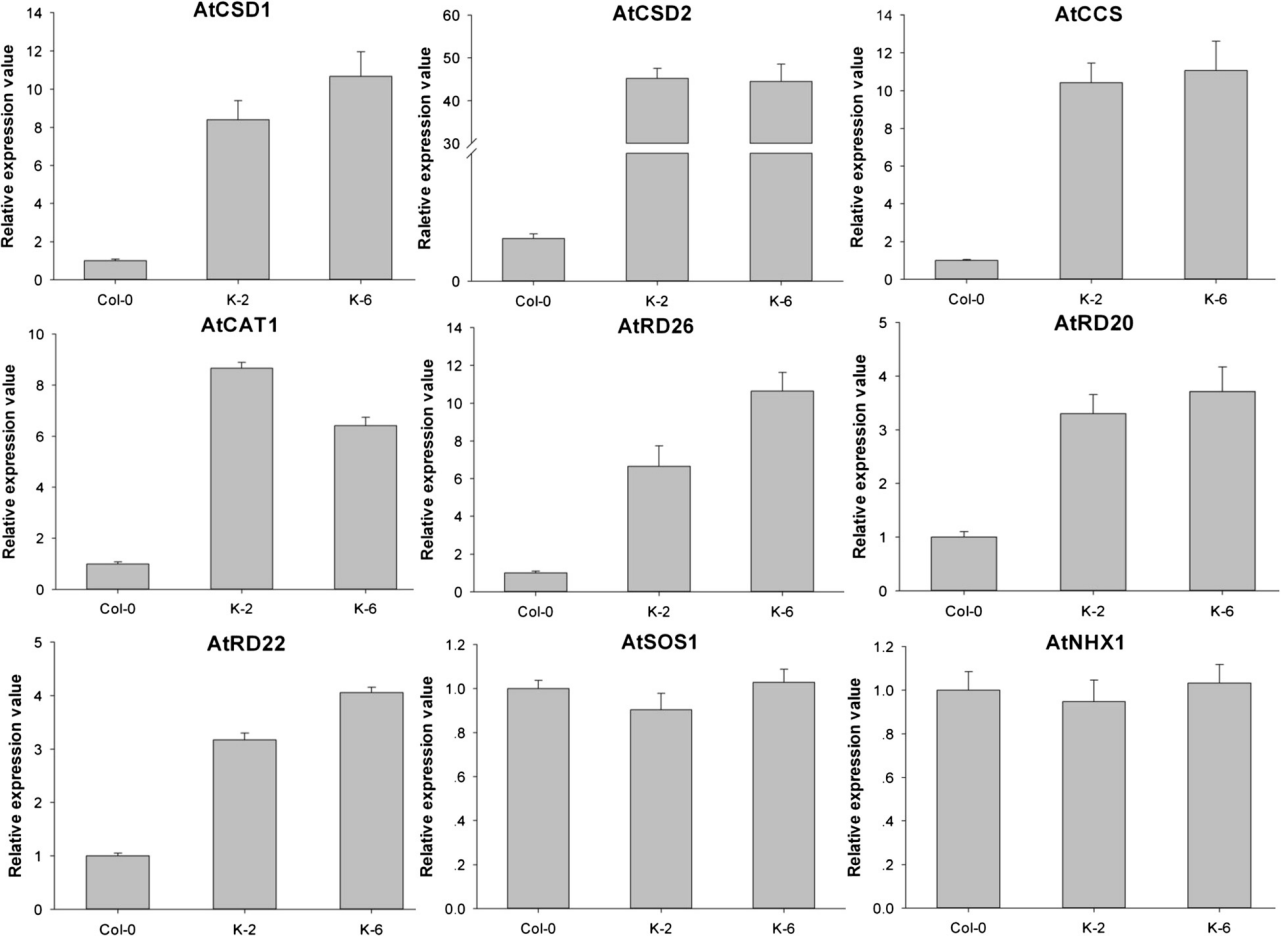
**The expression of stress-responsive genes in transgenic *GbRLK Arabidopsis *vs. nontransgenic Col-0.** Relative expression levels of stress-responsive genes were determined by qRT-PCR using cDNA synthesized from total RNAs isolated from the leaves of 2-week-old *Arabidopsis* grown in soil under normal conditions. Relative gene expression levels were determined using the 2^-ΔΔCT method. The CT (cycle threshold) values for both the target and internal control genes were means of three technical replicates. ΔCt = Ct target gene-CtRuBisCo. ΔΔCT = (CTtarget -CTRuBisCo)Transgenic - (CTtarget - CTRuBisCo) Non-transgenic. The large subunit of the RuBisCo gene (AtRuBisCo-F3/R3; Additional file 1: Table S1) from *Arabidopsis* was used as an internal control for normalization of different cDNA samples. The primers for the amplification of stress-responsive genes (Additional file 1: Table S1) were design based on sequences obtained from NCBI using Primer 5.0 software. Error bars represent standard error of means based on three independent reactions.

To reveal the possible molecular mechanisms underlying the improvement of stress resistance and tolerance in transgenic *Arabidopsis thaliana* by *GbRLK*, we studied the expression patterns of stress-responsive genes (*AtRD20, AtRD22* and *AtRD26*), ion transporter genes (*AtNHX1* and *AtSOS1*) and antioxidant genes (*AtCAT1, AtCCS, AtCSD2* and *AtCSD1*) in transgenic and nontransgenic *Arabidopsis thaliana*. We examined the expression of these genes in three *Arabidopsis* lines, including two genetically pure clones, K-6 and K-2, which exhibited differences in stress resistance, and the nontransgenic line Col-0. The expression of all of the genes, except for ion transporter genes (*AtNHX1, AtSOS1*), was upregulated in transgenic *GbRLK* plants compared with Col-0 plants, especially antioxidant genes (*AtCAT1, AtCCS*, *AtCSD2* and *AtCSD1*), which exhibited higher levels of upregulation than other genes (Figure 6).

## Discussion

Since RLKs were first reported, many kinases that play important roles in plant responses to abiotic stress and the ABA signaling network have been identified [27,28]. We have identified a RLK gene, *GbRLK* from cotton. The deduced protein sequence analysis of this gene revealed the presence of high structural homology with RLK orthologs from other plants. The results of phylogenetic analysis suggested that GbRLK protein was clustered together with other reported receptor-like kinase proteins and displayed the close phylogenetic relationship with Arabidopsis protein AT5G2408 and AT4G32300, which up-regulated with drought treatment [24-26]. This result indicated its potential function on abiotic stresses.

Interestingly, we found the increased expression of *GbRLK* transcript was observed under ABA, drought and high salt treatment, as shown by qRT-PCR, with highest early induction (4 h) found by ABA, suggesting the stress responsive transcript accumulation of *GbRLK* in cotton. Moreover *GbRLK* transcription was first transiently induced by ABA, while transcription induced by NaCl and drought treatment occurred later. In addition, ABA treatment induced higher levels of upregulated GUS expression than NaCl or drought, with NaCl producing the least upregulated GUS expression. We therefore conclude that *GbRLK* is a member of the ABA signal transduction pathway and activates ABA signal transduction. Drought or high-salt treatment increases ABA levels, which in turn results in the up-regulation of *GbRLK*. Meanwhile, overexpression of *GbRLK* can increase drought and high-salt tolerance in transgenic *Arabidopsis.* These results indicate that this *RLK* gene from cotton may likely function as a component of a signaling pathway that confers tolerance to abiotic stresses. *GbRLK* may play an important role in abiotic stress responses and the ABA signaling network.

ABA plays an important role in the tolerance of plants to abiotic stresses. ABA-independent and ABA-dependent signal transduction cascades are involved in the inducible expression of specific genes by abiotic stresses [36]. ABRE, MYB and MYC recognition sites function in ABA-dependent pathways 38]. The analysis of the promoters of such ABA-regulated genes has revealed a conserved *cis*-element, designated ABA-responsive element (ABRE; PyACGTGGC), which controls ABA-regulated gene expression [37]. In the ABA-independent pathway, gene expression in response to drought is regulated through DREs/CRT cis-acting elements or through NAC recognition sites [38]. Based on the current study, the *GbRLK* promoter contains two ABREs and some MYB and MYC-core recognition sites and does not contain DREs/CRT cis-acting elements or an NAC recognition site. Moreover, the *GbRLK* promoter and the *GbRLK* gene can respond to ABA, drought and high-salt stress. This study revealed a strong correlation between the inducibility of the *GbRLK* promoter and its internal *cis*-elements. For example, the W-box [39] and ABRE motifs [37,38], which are defense-responsive elements and *cis*-acting elements involved in abscisic acid responsiveness, may be the regulatory motifs of the *GbRLK* promoter that are responsible for the plant responses to the defense-related stimuli of *V. dahliae* and/or to ABA, PEG and NaCl. This information, combined with the results of the present study, suggests that *GbRLK* may take part in the ABA-dependent signaling pathway.

Water loss in plants is caused by drought and high-salinity conditions. ABA regulates stomatal opening in plants to avoid the affects of water loss due to drought and high salinity [40]. In this study, the water loss rate of the transgenic lines was significantly lower than that of the WT control. Thus, we can speculate that the improvement of resistance to drought and salt stresses may result from the ABA signaling pathway activated by the *GRLK* gene, which regulated other genes expression to decrease transpiration and water loss.

By contrast, the expression levels of two stress-responsive ion transporter genes, *AtNHX1*[41,42] and *AtSOS1*[42,43], were not significantly different in transgenic and nontransgenic *Arabidopsis.* In addition, *GbRLK* expression increased in response to *Verticillium dahliae* infection [32]. This pathogen promotes lignification of the cell wall, the formation of lignitubers and the restriction of vascular spread [44]; these responses not only prevent the pathogen from spreading further, but they also restrict water transport within the vascular system and alter the infected plant’s water balance or flux, causing severe dehydration stress. These results indicate that *GbRLK* is more highly induced by water deficit than by osmotic stress. Therefore, the main effect of the overexpression of *GbRLK* is a reduction in the amount of water loss in drought- and high-salt-stressed plants.

The molecular mechanisms underlying the response of higher plants to drought and high-salt stress are complicated. In response to these stress factors, various genes are up-regulated, which can mitigate the effects of stress and lead to an adjustment of plant stress tolerance. *RD20* is one of the most highly stress-responsive genes and is often used as a stress marker gene [45-47]. RD26, which encodes an NAC protein, is induced by drought, high salinity and ABA and is probably involved in a novel ABA-dependent stress-signaling pathway [48]. *RD22* is another stress-responsive gene [49]. In the current study, the increased expression of *RD22*, *RD20* and *RD26* in *GbRLK* plants compared with WT plants further supports the hypothesis that *GbRLK* plays a role in dehydration-stress tolerance.

The generation of reactive oxygen species (ROS) is common component of plant responses to abiotic stress and ABA [50]. Plants have evolved a complex antioxidant system to detoxify stress-induced ROS, in which ROS scavenging enzymes such as superoxide dismutase play essential roles [51]. The increased expression of *AtCSD1*[52], *AtCSD2*[52], *AtCCS* and *AtCAT1*[53] in *GbRLK* transgenic plants that was observed in the current study may have led to the scavenging of ROS produced under drought stress. These observations have clearly demonstrated the indirect role that *GbRLK* plays in protecting plants from dehydration stress by scavenging oxygen radicals. This study further clarifies the role played by *GbRLK* in improving stress tolerance in plants by affecting different stress-related pathways.

## Conclusions

This study demonstrates that the manipulation of a *GbRLK* gene from cotton using a transgenic approach can lead to improved high-salt and drought-stress tolerance in plants. We also obtained similar results by analyzing the promoter of the *GbRLK* gene. Thus, we hypothesize that *GbRLK* activates or participate in the ABA signaling pathway, and overexpression of *GbRLK* may improve stress tolerance by regulating stress-responsive genes to reduce the damage caused by water loss. The results of this study help clarify the role of *GbRLK* in improving stress tolerance in plants by affecting different stress-related pathways. The *GbRLK* gene will be a candidate gene for future research on abiotic stress signaling pathways and the genetic engineering of novel cotton cultivars.

## Methods

### Plant and fungi material, growth conditions

Cotton seeds of *G. barbadense* L. cv. Hai7124, a *Verticillum* wilt*-*resistant cultivar widely used in China for genetic and breeding studies, were delinted with concentrated sulfuric acid. To ensure that the seeds were free of pathogens, the seeds were subjected to 37% formaldehyde fumigation for 24 h. The treated cotton seeds were planted in pots filled with sterile soil using one seed per pot in growth chambers at 27°C 16-h light and 22°C 8-h dark regimen. The relative humidity was approximate 70% (± 5%).

*Arabidopsis* seeds were surface-sterilized with a solution of NaClO (0.5%, w/v) and Triton X-100 (0.01%, v/v) for 10 min followed by four rinses with sterile water. The seeds were placed into Petri dishes containing MS agar (0.8%, w/v) medium and incubated for 2 days at 4°C, followed by transfer to 22°C for germination. After 7 days of growth on plates, the seedlings were transplanted to pots containing soil mixture (rich soil:vermiculite = 2:1, v/v) and maintained in growth chambers at 22°C under a 16-h light/ 8-h dark regimen. The relative humidity was approximate 70% (± 5%).

*V. dahliae* cultured on PDA medium (200 g potato, 17 g agar, 20 g sucrose, 1,000 ml H_2_O) was used to inoculate 100 ml Czapek’s liquid medium (2 g NaNO_3_, 1 g K_2_HPO_3_, 0.5 g MgSO_4_ · 7H_2_O, 0.5 g KCl, 0.01 g FeSO_4_ · 7H_2_O, 30 g sucrose, 1,000 ml distilled H_2_O) in a 250-ml flask, which was cultured at 25°C for 8–10 d with shaking. The *V. dahliae* culture was then passed through four layers of cloth, and the number of spores was counted under a microscope. The final spore concentration was adjusted to 1 × 10^8^ immediately prior to plant inoculation.

### Stress treatment

*G. barbadense* L. cv. Hai7124, seedlings containing two simple leaves and one heart-shaped leaf were subjected to various stress treatments. ABA treatment was carried out by spraying the plants with a 200 μM ABA solution at 0, 1, 2, 4, 6, 8, 10 and 12 h. Dehydration stress and salt-stress treatments were performed by submerging the roots of the plants in 20% PEG6000 or 200 mM NaCl solution under the same temperature and light conditions for 0, 1, 2, 4, 8, 12 and 24 h. Low-temperature stress was applied by transferring the plants to a growth chamber set at 4°C under the same photoperiodic conditions at 0, 1, 2, 4, 8, 12 and 24 h. SA and MeJA treatment were carried out by spraying the plants with 2 mM SA and 100 μM MeJA solution at 0, 2, 4, 6, 8, 10 and 12 h. After exposure to stress, the cotton leaves were immediately frozen in liquid nitrogen prior to expression analysis.

The seedlings were analyzed for ABA sensitivity as described by Fujita [48]. To test the phenotypes of plants grown in soil under salt and drought stress, *Arabidopsis* seedlings grown on MS medium for seven days were transferred to mixed soil (rich soil:vermiculite = 2:1, v/v) and grown for two week with sufficient watering. Each pot contained five seedlings. To investigate the tolerance to drought and high salt stresses, we supplied enough and equal volume water for every pot plants at the beginning of withholding irrigation and submerging the roots of the plants in 200 mM NaCl solution. The plants were then subjected to drought stress treatment by withholding irrigation for 42 days; the plants were grown at 22°C under a 16-h light/8 h dark regimen. For salt-stress analysis, wild-type and transgenic plants grown in pots at 22°C under a 16-h light/8 h dark regimen were irrigated with 200 mM NaCl solution once a week for 5 weeks. All pots were placed under the same conditions in a growth chamber in random order, and the position of each pot was randomly changed every day to exclude “positional effects”. The experiments were repeated for three times.

To measure GUS activity, *Arabidopsis* plants grown on MS medium for 14 days were transferred to one of the following solutions: 100 μM ABA, 20% PEG6000, 200 mM NaCl or 1 × 10^8^ spore/ml *V. dahliae* spore suspension for 4 h, 12 h, 12 h or 96 h. After exposure to stress, the *Arabidopsis* seedlings were immediately frozen in liquid nitrogen prior to GUS activity analysis.

To measure germination rates and the rate of chlorosis of the transgenic lines, we planted *Arabidopsis* plants on MS medium containing different concentration NaCl for 14 days, then gathered the data.

### Cloning the *GbRLK* promoter

Self-formed adaptor PCR (SEFA-PCR) was performed to clone the promoter sequence of *GbRLK*[54]. The detailed procedures were as follows: long and accurate Taq, buffer and deoxynucleoside triphosphates were purchased from TaKaRa Biotechnology Co., Ltd (Shiga, Japan). The PCR mixture included 15 μl of 2 × GC buffer I, 5 μl of 2.5 mM deoxynucleoside triphosphates, 1.5 U of long and accurate Taq enzyme and approximately 1 μg of H7124 template genomic DNA; deionized water was added to a final volume of 30 μl. PCR amplification was performed using three gene-specific internal primers, including SP1, SP2 and SP3 (Additional file 1: Table S1).

### PCR amplification

Standard polymerase chain reaction (PCR) was completed using rTaq DNA polymerase (TaKaRa Biotechnology (Dalian) Co., Ltd., China). The reaction system of PCR products contain 2 μl 10 × buffer, 1 μl Mg^2+^, 0.2 μl rTaq DNA polymerase, 1 μl template and 0.5 μl dNTP in 20 μl volume to PCR thermal cycling conditions were: 4 min at 94°C; 30 cycles of 30s at 94°C, 30 s at 55-62°C, 0.5-1.5 min at 72°C; and finally 7 min at 72°C [55]. The annealing temperature and extending time were changed based on the different primers. The primers used in this paper were listed in Additional file 1: Table S1.

### Plasmid construction and *Arabidopsis* transformation

To construct the 35S::*GbRLK* vector, the entire coding region of *GbRLK* was amplified by PCR using *Sma* I and *Xba* I linker primers (GbRLK1-F/R) (Additional file 1: Table S1) and ligated into vector pBI121. To construct the p*GbRLK*::GUS vector, a 1,890-bp fragment of the *GbRLK* promoter was chosen. This fragment was amplified by PCR using *EcoR* I and *Bgl* II linker primers (pGbRLK1-F/R; Additional file 1: Table S1) and ligated into vector pCAMBIA1301. Specifically, the PCR-amplified fragment was digested with *EcoR* I and *Bgl* II (Promega, Madison, WI, USA) and purified with a TIAN-quick Midi Purification Kit (TIANGEN, Beijing, People’s Republic of China). The fragment was then fused to the GUS reporter gene of the pCAMBIA1301 vector, which was previously digested with *EcoR* I and *Bgl* II to release the 35S promoter. The resulting vector, named P-1890/GUS, was confirmed by sequencing; the pCAMBIA1301 vector served as a control and was named 35S/GUS.

The constructs were introduced into *Agrobacterium tumefaciens* (strain GV3101) and transferred into *Arabidopsis* (Col-0) using the floral dip method [56]. Transgenic *GbRLK* plants were confirmed by examining the segregation ratio of the *kanamycin* selectable marker and by PCR analysis of *NPT*II and 35S-*GbRLK* using the primers NPTII-F/R and 35S-GbRLK (Additional file 1: Table S1). The primers GbRLK2-F/R (Additional file 1: Table S1) were designed to evaluate the expression level of *GbRLK* in *Arabidopsis* by qRT-PCR. Southern blotting analysis of transgenic *Arabidopsis* plants was conducted using the 3′ 407-bp sequence of the full-length cDNA, which was amplified using GbRLK3-F/R primers (Additional file 1: Table S1), as a probe. Hybridization was conducted according to the instructions of the DIG High Prime DNA labeling and Detection Kit (Roche, Switzerland). Transgenic *Arabidopsis* plants with P-1890/GUS were confirmed using primers designed from the promoter sequence (pGbRLK1-F/R) and *GUS* (GUS-F/R; Additional file 1: Table S1)*.* The transgenic lines used for analysis in this study were T5 homozygous plants.

### Quantitative real time reverse transcription polymerase chain reaction (qRT-PCR)

Total RNA was isolated using methods developed from the cetyltrimethyammonium brodmide (CTAB) method [57]. First-strand cDNA was synthesized from 1 μg total RNA using the M-MLV reverse transcriptase (Promega, USA) according to the manufacturer’s instructions. Quantitative PCR (qRT-PCR) was used in order to evaluate the expression levels of *GbRLK* gene under different stress treatments using the leaves of *G. barbasense* L. cv. Hai7124 and the ABI 7500 Real Time System (PE Applied Biosystems, USA). Gene-specific primers were designed according to *GbRLK* sequences using Primer Premier 5.0 (Additional file 1: Table S1) and synthesized by GenScript Corp (Nanjing, China). The transcript encoding cotton elongation factor (*EF1α*) was amplified as a standard control for qRT-PCR analysis of cotton, and the large subunit of the RuBisCo gene (AtRuBisCo-F3/R3; Additional file 1: Table S1) [58] from *Arabidopsis* was used as an internal control for normalization of different *Aarabidopsis* cDNA samples Melt curve analysis was performed to check the specificity of amplified product and relative gene expression levels were determined using the 2-ΔΔCT method [59]. The CT (cycle threshold) values for both the target and internal control genes were means of three technical replicates. ΔCt = Ct target gene-Ct EF1α. ΔΔCT = (CTtarget - CT control)treatment/transgenic - (CTtarget - CT control)control.

### Measurement of transpiration rates

For water loss measurement, rosette leaves were detached from 4-week-old plants and weighted immediately on a piece of weighting paper, which was then placed on a laboratory bench (at a relative humidity of 40% to 50% and a temperature of 22°C to 23°C). The weight loss of each sample was measured at designated time points. The proportion of water loss was calculated on the basis of the initial fresh weight of the sample; water loss was expressed as a percentage of the initial fresh weight. All tests were repeated at least three times.

### Histochemical and fluorometric GUS assay

For histochemical staining of GUS, fresh tissue samples were obtained from *Arabidopsis* plants and immediately exposed to X-Gluc solution. After overnight incubation at 37°C, stained samples were bleached with 70% (v/v) ethanol and observed under OLYMPUS BX51 and SZX12 microscopes. A fluorometric GUS assay was performed as described by Jefferson *et al.*[60].

The fluorescence of 4-methylumbelliferone (4-MU), the product of GUS-catalyzed hydrolysis, was measured using the TECAN CENios system. The protein concentration in the supernatant was assessed by the Bradford [61] method, using bovine serum albumin (BSA) as a standard. GUS activity was normalized to the protein concentration of each supernatant extract and calculated as pmol of 4-MU per milligram of soluble protein per minute.

### Measurement of anthocyanin and chlorophyll content

Anthocyanin was extracted and quantified as described by Gareth et al*.*[62] from shoot tissues of 10–15 plantlets 3 weeks after treatment with salt and drought*.* Results are expressed as absorbance at 530 nm with a spectrophotometer (Shimadzu UV-1600, Japan). Chlorophyll was extracted and measured in triplicate as described by Lichtenthaler [63]. About 100 mg of fine powder of leaf tissue was homogenized in 1 ml of 80% acetone and kept for 15 min at room temperature in dark. The crude extract was centrifuged for 20 min at 10000 rpm (rotation per minute) at room temperature, and the resultant supernatant was used for assessing absorbance at 663 and 645 nm with a spectrophotometer (Shimadzu UV-1600, Japan). Chlorophyll and anthocyanin content were computed in terms of fresh weight (FW).

## Abbreviations

RLK: Receptor-like kinase; ABA: Abscisic acid; GUS: β-Glucuronidase gene; MS: Murashige and skoog medium; SEFA-PCR: Self-formed adaptor PCR; SA: Salicylic acid; JA: Jasmonic acid.

## Competing interests

The authors declare that they have no competing interests.

## Authors’ contributions

ZTZ designed the study and wrote the manuscript; ZJ performed the experiments and analyzed the data; GYL cloned the gene; ZZY measured the GUS activity; CTZ transformed the *Arabidopsis*; GWZ contributed reagents/materials/analysis tools. All authors read and approved the final manuscript.

## Supplementary Material

Additional file 1: Table S1 The sequences of primers employed in this study. Click here for file

Additional file 2: Figure S1 Cloning and analysis of *GbRLK* promoter. (a) Agarose gel electrophoresis of *GbRLK* promoter region by the Sefa -PCR method. Lane M: DNA Marker; Lane 1: the product of the first round of nested PCR; Lane 2: the product of the second round of nested PCR. (b) The sequence of the *GbRLK* promoter and cis-acting element analysis. The red background represents the initiation codon of *GbRLK*. The A nucleotide of the initiation codon ATG is numbered +1. The core promoters (TATA-box and CAAT-box) are indicated with a blue background, the putative transcriptional initiation site (TSS) A is shown in red and larger font and the cis-acting elements involved in light, regulation of plant growth and hormones are indicated by a boxed, yellow background. (c) *GUS* expression in P-1890/GUS transgenic *Arabidopsis* plants after treatment with different stressors. Click here for file

Additional file 3: Table S2 Cis-acting element analysis of 5′ flanking sequences of *GbRLK.* Click here for file

Additional file 4: Figure S2 Molecular analysis of independent transgenic *Arabidopsis* lines. (a) PCR tests of transgenic plants. (b) Southern blot analysis of four independent transgenic lines. P: positive control pBI121, N: non-transformed plant; Transgenic lines K-2, K-3, K-5 and K-6. (c) qRT–PCR analysis of *GbRLK* expression in homozygous transgenic *Arabidopsis* lines. The large subunit of the RuBisCo gene (AtRuBisCo-F3/R3; Additional file 1: Table S1) from *Arabidopsis* was used as an internal control for normalization of different cDNA samples. (d) Fresh weight of transgenic and nontransgenic *Arabidopsis* at different time points. Click here for file
