# Supplementary material for: A receptor-like kinase gene (GbRLK) from Gossypium barbadense enhances salinity and drought-stress tolerance in Arabidopsis
Source: BMC Plant Biol. 2013 Aug 6;13:110. doi: 10.1186/1471-2229-13-110 (PMC3750506; doi:10.1186/1471-2229-13-110)
Supplement: Additional file 1: Table S1 — The sequences of primers employed in this study. [file 1471-2229-13-110-S1.pdf]

Additional table 1 The sequence of primers

GbRLK1-F	CGTCTAGATAGTGTTTGCCCAGAAACTC
GbRLK1-R	ATCCCGGGTATTCTGGAAGCACATTA
GbRLK2-F	CAATAGGCACTGCTAAAGGG
GbRLK2-R	ACCACGATGCTCATCAAAGG
EF-F	AGACCACCAAGTACTACTGCAC
EF-R	CCACCAATCTTGTACACATCC
AtRuBisCo-F3	GCAAGTGTTGGGTTCAAAGCTGGTG
AtRuBisCo-R3	CCAGGTTGAGGAGTTACTCGGAATGCTG
GbRLK3-F	GCCAAGAATGGTTTCCAAGAT
GbRLK3-R	ACATTTTGTATTCTGGAAG CACA
NPTII-F	CACCCTATTCCC CTATCACTC
NPTII-R	TACAACCCCATCCCCTCCCA
pGbRLK1-F	CCGGAATTCTTTTAATTAGGGTAGACAG
pGbRLK1-R	GAAGATCTACCA TGTTTAGTGTTATAGATTGAG
35S-GbRLK	CACAATCCCACTATCCTTCG
GUS-F	GGTGGGAAAGCGCGTTACAAG
GUS-R	GTTTACGCGTGCTTCCGCCA
**The primers of sefa-PCR**	
SP1	GCAAACACTATCGCTGCTTTCTC
SP2	CCTGGTTGAACGGCTTAGTGA
SP3	TGATCACCTGTCCCTGGNNNNNNAAGA
**The primers of qRT-PCR (5—3)**	
AtRD22F	GATTCGTCTTCCTCTGATCTG
AtRD22R	TGGGTGTTAACGTTTACTCCG
AtNHX1F	CCGTGCATTACTACTGGAGACAAT
AtNHX1R	GTACAAAGCCACGACCTCCAA
AtCAT1F	AGCGCTTTCGGAGCCTCGTG
AtCAT1R	GGCCTCACGTTAAGACGAGTTGC
AtSOS1F	TCGTTTCAGCCAAATCAGAAAGT
AtSOS1R	TTTGCCTTGTGCTGCTTTCC
AtRD22F	GATTCGTCTTCCTCTGATCTG
AtRD22R	TGGGTGTTAACGTTTACTCCG
AtCDS2F	TTAGTCTGACCACTGGAAACGC
AtCDS2R	GGATGCTAAATAAACCAAAATGTA
AtCDS1F	GTTGGTAGGGCTGTTGTTGTC
AtCDS1R	TGGACCTCCTTATTACATCAA
AtCCSF	GGGTAGTCCGATTTGCTCAG
AtCCSR	GGTCTCCCAATGGCTCTGTG
AtRD20F	GTTTGGAATGTAACCGAGGGA
AtRD20R	AACTGAATAACAAGACGAAAGAAGC
AtRD26F	GATGTGAAGTTACTGATGGGTGAA
AtRD26R	GCGAGCCAAGTCACAAGGAG
