# Supplementary material for: A receptor-like kinase gene (GbRLK) from Gossypium barbadense enhances salinity and drought-stress tolerance in Arabidopsis
Source: BMC Plant Biol. 2013 Aug 6;13:110. doi: 10.1186/1471-2229-13-110 (PMC3750506; doi:10.1186/1471-2229-13-110)
Supplement: Additional file 3: Table S2 — Cis-acting element analysis of 5′ flanking sequences of GbRLK. [file 1471-2229-13-110-S3.pdf]

Additional table 2 Cis-acting elements analysis of 5’ flanking sequences in *GbRLK*

Classified of elements	Cis-acting elements	Sequence	Function
	MYCCONSENSUSAT	CATCTG	MYC recognition site found in the promoters of the dehydration responsive gene rd22
	MYB1AT	AAACCA	MYB recognition site found in the promoters of the dehydration responsive gene rd22
	2SSEEDPROTBANAPA	CAAACAC	Conserved in many storage protein gene promoters
	ACGTATERD1	ACGT	cis-acting elements of erd1, function in induction by dehydration stress
	AGMOTIFNTMYB2	AGATCCAA	AG-motif found at -114 of the promoter of NtMyb2 gene
	ABRE	CACGTG	cis-acting element involved in the abscisic acid responsiveness
	TCA-element	CCATCTTTTT	cis-acting element involved in salicylic acid responsiveness
	ARE	TGGTTT	cis-acting regulatory element essential for the anaerobic induction
**Elements related to stresses and hormone**	WBOXATNPR1	TTGAC	W-box found in promoter of Arabidopsis NPR1 gene
	WBOXHVISO1	TGACT	SUSIBA2 bind to W-box element in barley iso1 promoter
	WBOXNTERF3	TGACY	W box found in the promoter region of ERF3 gene in tobacco;
	WRKY71OS	TGAC	A core of TGAC-containing W-box
	MYBCORE	CNGTTR	Binding site for at least two plant MYB proteins ATMYB1 and ATMYB2
	RAV1AAT	CAACA	Binding consensus sequence of Arabidopsis transcription factor RAV1
	GT1CONSENSUS	GRWAAW	Consensus GT-1 binding site in many light-regulated genes
	EBOXBNNAPA	CANNTG	E-box of napA storage-protein gene of Brassica napus
	DOFCOREZM	AAAG	Core site required for binding of Dof proteins in maize
	TAAAGSTKST1	TAAAG	TAAAG motif found in promoter of Solanum tuberosum (S.t.) KST1 gene
